# Diabetes, obesity, hyperlipidemia, and sudden sensorineural hearing loss: A bidirectional and multivariable Mendelian randomization study

**DOI:** 10.1097/MD.0000000000048255

**Published:** 2026-04-10

**Authors:** Sufen Peng, Chunmei Lu, Li Lin, Jialei Chen, Chenghong Guo

**Affiliations:** a Department of Otorhinolaryngology, The People’s Hospital of Yubei District of Chongqing, Chongqing, China; b Department of Otolaryngology Head and Neck Surgery, Children’s Hospital of Chongqing Medical University, Chongqing, China.

**Keywords:** diabetes, hyperlipidemia, Mendelian randomization, obesity, sudden sensorineural hearing loss

## Abstract

Many compelling observational studies suggest a reasonable correlation between diabetes, obesity, hyperlipidemia, and susceptibility to sudden sensorineural hearing loss (SSNHL). Nevertheless, some studies have observed results that are entirely opposite to this. We conducted a bidirectional and multivariable Mendelian randomization (MR) analysis to determine whether those associations are causal. Independent genetic variants associated with diabetes, obesity, hyperlipidemia, and SSNHL at the genome-wide significance level were selected as instrumental variables. All summary data can be obtained from the genome-wide association study database. The primary method utilized in the MR analysis was the inverse variance weighted method, while sensitivity analyses employed the weighted median method, MR-Egger method, and MR-PRESSO method. The MR analysis conducted using the inverse variance weighted method reveals a potential causal link between type 2 diabetes and the risk of SSNHL (odds ratio = 1.18, 95% confidence interval = 1.02–1.36, *P* = .03). These findings remain consistent in multivariate MR analysis, even after adjusting for obesity and hyperlipidemia (odds ratio = 1.17, 95% confidence interval = 1.01–1.36, *P* = .03). However, there is no discernible genetic evidence supporting a direct causal link between type 1 diabetes, obesity, and hyperlipidemia and the occurrence of SSNHL. Our bidirectional and multivariable MR analysis indicates that type 2 diabetes is a potential risk factor for SSNHL. Meanwhile, no direct causal relationship is identified between type 1 diabetes, obesity, hyperlipidemia, and the risk of SSNHL.

## 1. Introduction

Sudden sensorineural hearing loss (SSNHL) is characterized by an abrupt onset of hearing impairment, typically evolving over a short period. It was defined as a sudden occurrence of unexplained sensorineural hearing loss occurring over a 72-hour timeframe, encompassing a hearing threshold loss >30 dB across at least 3 consecutive frequencies.^[1]^ Patients may experience sudden sensations of ear fullness or blockage, accompanied by a decline in hearing acuity.^[2]^ The incidence of SSNHL is relatively low, ranging from 5 to 400 cases per 100,000 individuals annually worldwide.^[3]^ However, the global incidence of SSNHL is gradually increasing.^[4]^ Proposed mechanisms include inflammation,^[5]^ viral infections,^[6]^ autoimmune diseases,^[7]^ or inadequate blood supply to the inner ear.^[8]^ Notably, growing evidence underscores inflammation as a critical contributor to its pathogenesis, with observed inflammatory cytokine infiltration in the cochlea of affected patients.^[9]^

Concurrently, metabolic disorders such as type 2 diabetes (T2D), obesity, and dyslipidemia have been frequently implicated as potential risk factors for SSNHL in observational studies.^[10,11]^ A compelling pathophysiological link between these conditions and SSNHL may lie in their shared characteristic of a chronic inflammatory state. Obesity is recognized as a state of systemic meta-inflammation, where adipose tissue dysfunction drives the continuous release of pro-inflammatory cytokines.^[12,13]^ Similarly, dyslipidemia is not merely a lipid imbalance but is closely associated with elevated inflammatory biomarkers, contributing to endothelial dysfunction and atherosclerosis.^[14,15]^ Furthermore, chronic inflammation is a cornerstone in the development and progression of T2D, inducing insulin resistance and pancreatic β-cell damage.^[16,17]^ This systemic inflammatory burden can compromise the delicate microenvironment of the inner ear. The cochlea, particularly the stria vascularis responsible for maintaining the endocochlear potential, is highly vulnerable to vascular and inflammatory insults.^[18]^ Inflammation can disrupt the blood-labyrinth barrier, impair microcirculation, and directly damage hair cells, thereby providing a plausible biological mechanism linking these metabolic conditions to SSNHL.^[19]^ Despite these plausible connections, findings from observational studies remain inconsistent. Some reports indicate a significantly higher incidence of SSNHL in individuals with T2D, obesity, or dyslipidemia,^[11,20]^ while others find no significant association after adjusting for confounders like age and cardiovascular disease.^[21–23]^ These discrepancies likely stem from inherent limitations of observational designs, including residual confounding, reverse causality, and selection bias.

To overcome these methodological constraints, Mendelian randomization (MR) has emerged as a powerful tool that uses genetic variants as instrumental variables (IVs) to infer causal relationships.^[24]^ Previous studies utilizing MR analysis have successfully confirmed causal relationships between inflammatory factors,^[5]^ thyroid function,^[3]^ and blood lipids^[25]^ with SSNHL. Building upon this foundation, our study employs large-scale genome-wide association studies (GWAS) and utilizes bidirectional MR and multivariable MR (MVMR) analyses to examine the causal relationships and risks between diabetes, obesity, hyperlipidemia, and SSNHL. By doing so, we aim to provide new perspectives and insights into the etiology of SSNHL and develop effective prevention and treatment strategies for the benefit of SSNHL patients.

## 2. Method

### 2.1. Study design

**Figure 1.**
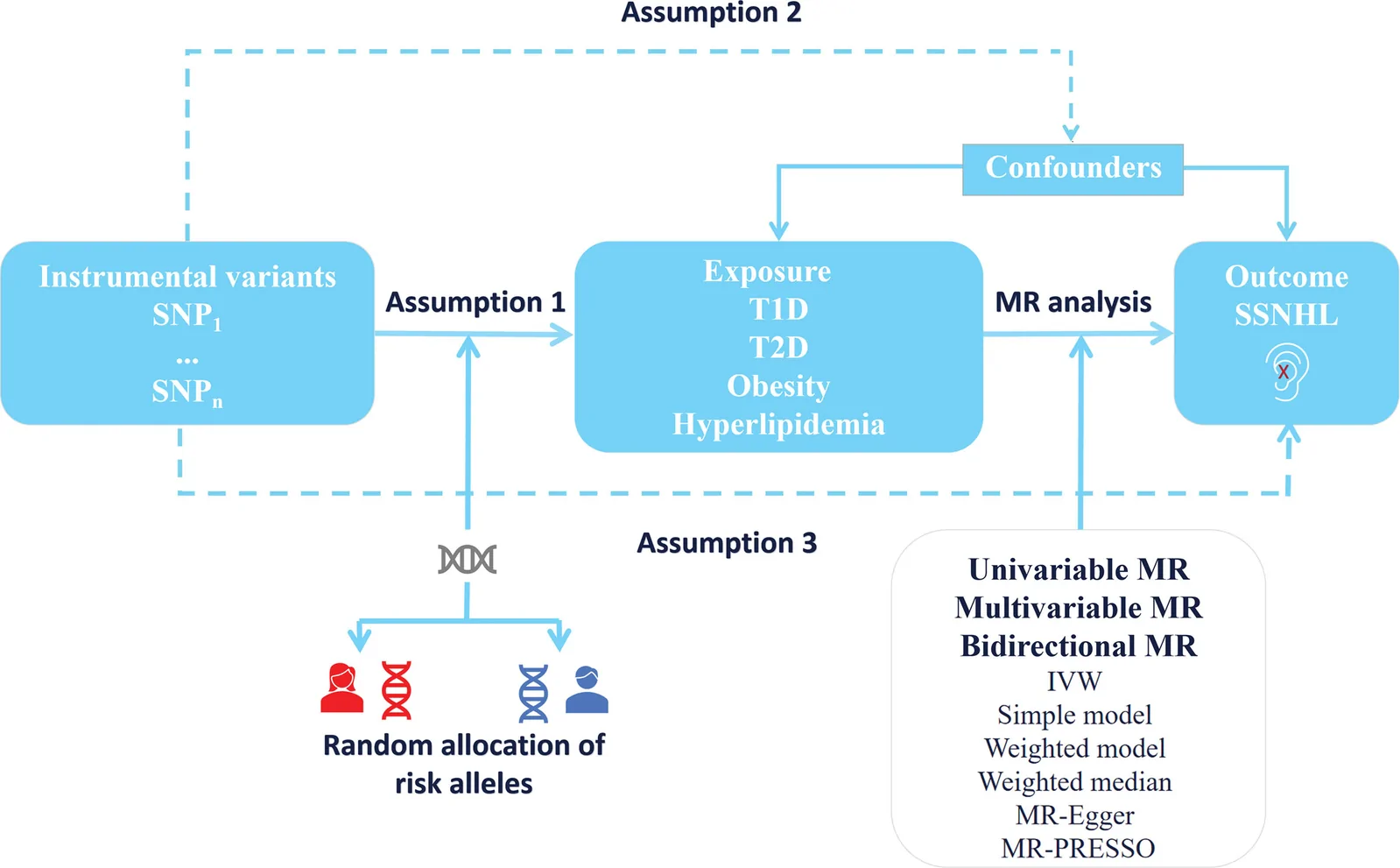
A framework design for bidirectional and multivariable MR analyses. Assumption 1: genetic variants are associated with exposure. Assumption 2: genetic variants are not associated with any known or unknown confounders. Assumption 3: genetic variants should only affect the risk of outcome through exposure. IVW = inverse variance weighted, MR = Mendelian randomization, SNP = single-nucleotide polymorphism, SSNHL = sudden sensorineural hearing loss, T1D = type 1 diabetes, T2D = type 2 diabetes.

Figure 1 presents an overview of the study design, illustrating the framework utilized in the research. In essence, we conducted bidirectional MR and MVMR studies using GWAS data on diabetes, obesity, hyperlipidemia, and SSNHL. To minimize population stratification bias, both exposure and outcome cohorts were restricted to individuals of European ancestry. A robust MR design relies on 3 fundamental assumptions: the relevance assumption posits a strong correlation between genetic variants and the exposure factor (in this case, diabetes, obesity, and hyperlipidemia); the independence assumption assumes that genetic variants are independent of confounding factors that could potentially influence both the exposure and the outcome; and the exclusion restriction assumption suggests that genetic variants affect the outcome (especially SSNHL) solely through the exposure and not through alternative pathways.^[26]^ Given that this study involves a reanalysis of previously published data, no additional ethical approval is required.

### 2.2. GWAS data of diabetes, obesity, hyperlipidemia, and SSNHL

The genetic data related to diabetes, obesity, and hyperlipidemia were obtained from the GWAS catalog (https://gwas.mrcieu.ac.uk/). The GWAS ID for type 1 diabetes (T1D) is “finn-b-T1D_WIDE,” encompassing 189,302 participants (6729 cases and 182,573 controls). The GWAS ID for T2D is “finn-b-T2D_WIDE,” encompassing 202,046 participants (17,268 cases and 184,778 controls). The GWAS ID for hyperlipidemia is “finn-b-E4_HYPERLIPNAS,” encompassing 209,714 participants (4535 cases and 197,259 controls). The GWAS ID for obesity is “finn-b-E4_OBESITY,” encompassing 218,735 participants (8908 cases and 209,827 controls). The GWAS ID for SSNHL is “finn-b-H8_HL_IDIOP,” encompassing 196,592 participants (1491 cases and 195,101 controls).

### 2.3. IV selection

Based on GWAS results for T1D, T2D, obesity, and hyperlipidemia, single-nucleotide polymorphisms (SNPs) closely associated and reaching genome-wide significance (*P* < 5 × 10^−8^) were carefully screened. SNPs showing a strong association with SSNHL were defined as having a significance level of *P* < 5 × 10^−6^.^[5]^ These selected SNPs were subsequently employed as IVs in MR analysis. The criteria for IVs selection in MR analysis were as follows: to mitigate estimation bias arising from weak IVs, the equation *F* = (*R*^2^ × (n − 2))/(1 − *R*^2^) was utilized to assess the correlation between instrument strength and exposure. A significant correlation was considered when *F* ≥ 10. The estimated *R*^2^ for IVs was calculated using the equation 2EAF(1 − EAF) × β^2^, where EAF represents the frequency of the effect allele, and β represents the estimated genetic effect on exposure factors.^[3]^ To obtain independent IVs, we performed clumping (*R*^2^ < 0.001 within a 1000-kb distance) based on the linkage disequilibrium reference panel of the 1000 Genomes Project while excluding palindromic SNPs with moderate allele frequencies.^[27]^ In accordance with the exclusion restriction assumption (IVs only affecting SSNHL through exposure), all SNPs associated with hearing loss (*P* < 1 × 10^−5^) were excluded from each analysis.^[5]^ The PhenoScanner database (https://www.repository.cam.ac.uk/items/31e4df31-982b-452e-baf8-83ce942b1c0a) was used to exclude all known phenotypes associated with any genetic instruments considered in our analysis.^[28]^ Table S1, Supplemental Digital Content, https://links.lww.com/MD/R616, provides a comprehensive summary detailing the relationship between exposure, SNPs, and their associations with outcomes.

### 2.4. Univariate MR analysis

Using the inverse variance weighted (IVW) method as the primary analysis, we employed a series of complementary MR tests to rigorously assess causal effects and correct for the impact of horizontal multiplicity.^[29]^ In summary, additional sensitivity analyses were conducted using MR-Egger^[30]^ and weighted median^[31]^ as complementary methods to IVW. The MR-PRESSO method aimed to identify and eliminate outliers, producing relatively unbiased estimates while also detecting potential horizontal pleiotropy effects through global tests.^[32]^ The Cochran *Q* test was utilized to assess SNP heterogeneity, where a *P* value >.05 indicated no heterogeneity. In addition, leave-one-out analysis was performed, systematically removing 1 SNP at a time to assess whether bias in MR estimates was driven by individual SNPs.^[33]^ Using the same methods, a reverse MR analysis was conducted to explore the possibility of SSNHL as a risk factor for diabetes, obesity, and hyperlipidemia. Given that genotypes are determined at conception according to Mendel law of segregation, the likelihood of reverse causality is significantly reduced.^[34]^

### 2.5. MVMR analysis

Previous observational studies have indicated that obesity, hyperlipidemia, and diabetes are significant risk factors for the occurrence of SSNHL. Therefore, we employed MVMR-IVW as the primary analytical method for conducting MVMR analysis, aiming to identify independent risk factors influencing SSNHL. We assessed instrument strength in the multivariable context using conditional *F*-statistics, with values >10 indicating adequate strength. Multicollinearity was evaluated using variance inflation factors, with values exceeding 10 suggesting potentially problematic collinearity. All analyses were performed using R software (version 4.2.2; MRC Integrated Epidemiology Unit [MRC IEU], University of Bristol, Bristol, United Kingdom) with the “MendelianRandomization,” “TwoSampleMR,” “MRPRESSO,” and “MVMR” packages for MR statistical analysis and data visualization. Statistical significance in estimating the causal effect of exposure was determined with *P* values <.05.

## 3. Result

### 3.1. Univariate MR analysis of the causal relationship between diabetes, obesity, hyperlipidemia, and SSNHL

**Figure 2.**
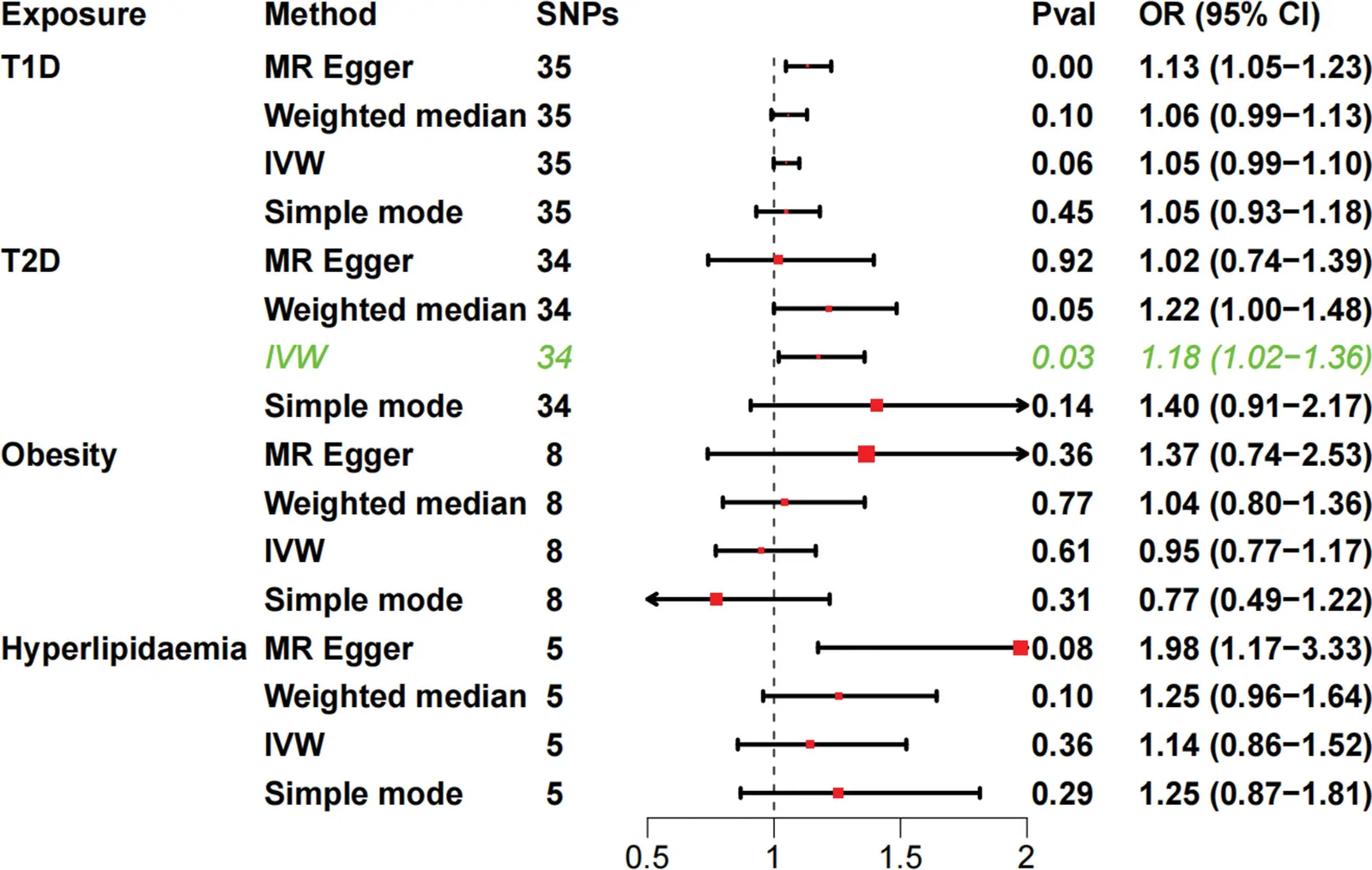
Univariate MR analysis of the causal relationship between diabetes, obesity, hyperlipidemia, and SSNHL. CI = confidence interval, IVW = inverse variance weighted, MR = Mendelian randomization, OR = odds ratio, SNP = single-nucleotide polymorphism, SSNHL = sudden sensorineural hearing loss, T1D = type 1 diabetes, T2D = type 2 diabetes.

**Figure 3.**
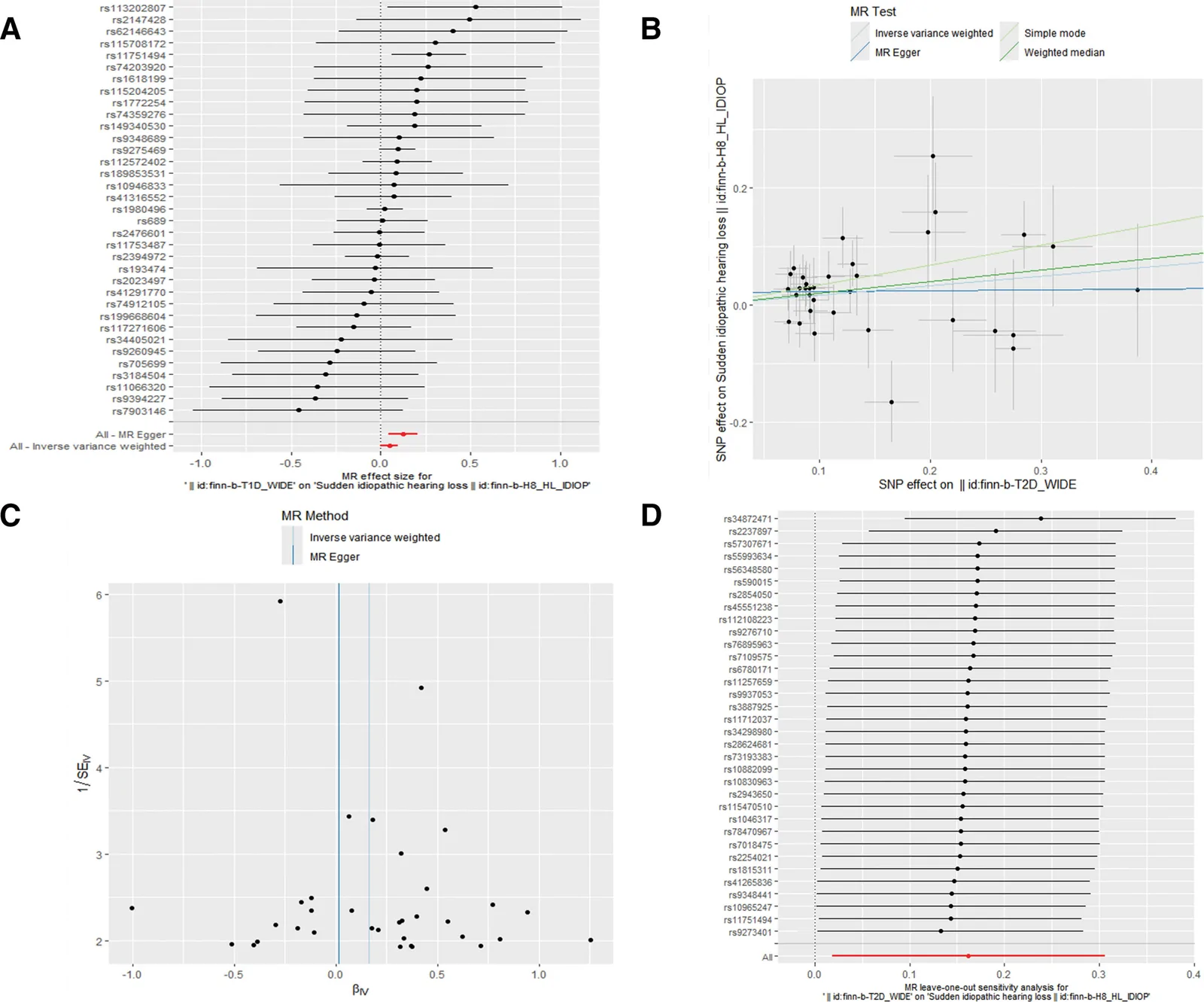
Univariate MR analysis of the causal relationship between diabetes, obesity, hyperlipidemia, and SSNHL. (A) Forest plot of the potential effects of T2D-associated SNPs on SSNHL. (B) Scatter plot demonstrates the effect of each T2D-associated genetic variant on SSNHL on the log-odds scale. (C) Funnel plot of the causal effect of T2D-related SNPs on SSNHL. (D) Leave-one-out plots for the MR analyses of SSNHL on T2D. MR = Mendelian randomization, SNP = single-nucleotide polymorphism, SSNHL = sudden sensorineural hearing loss, T2D = type 2 diabetes.

**Figure 4.**
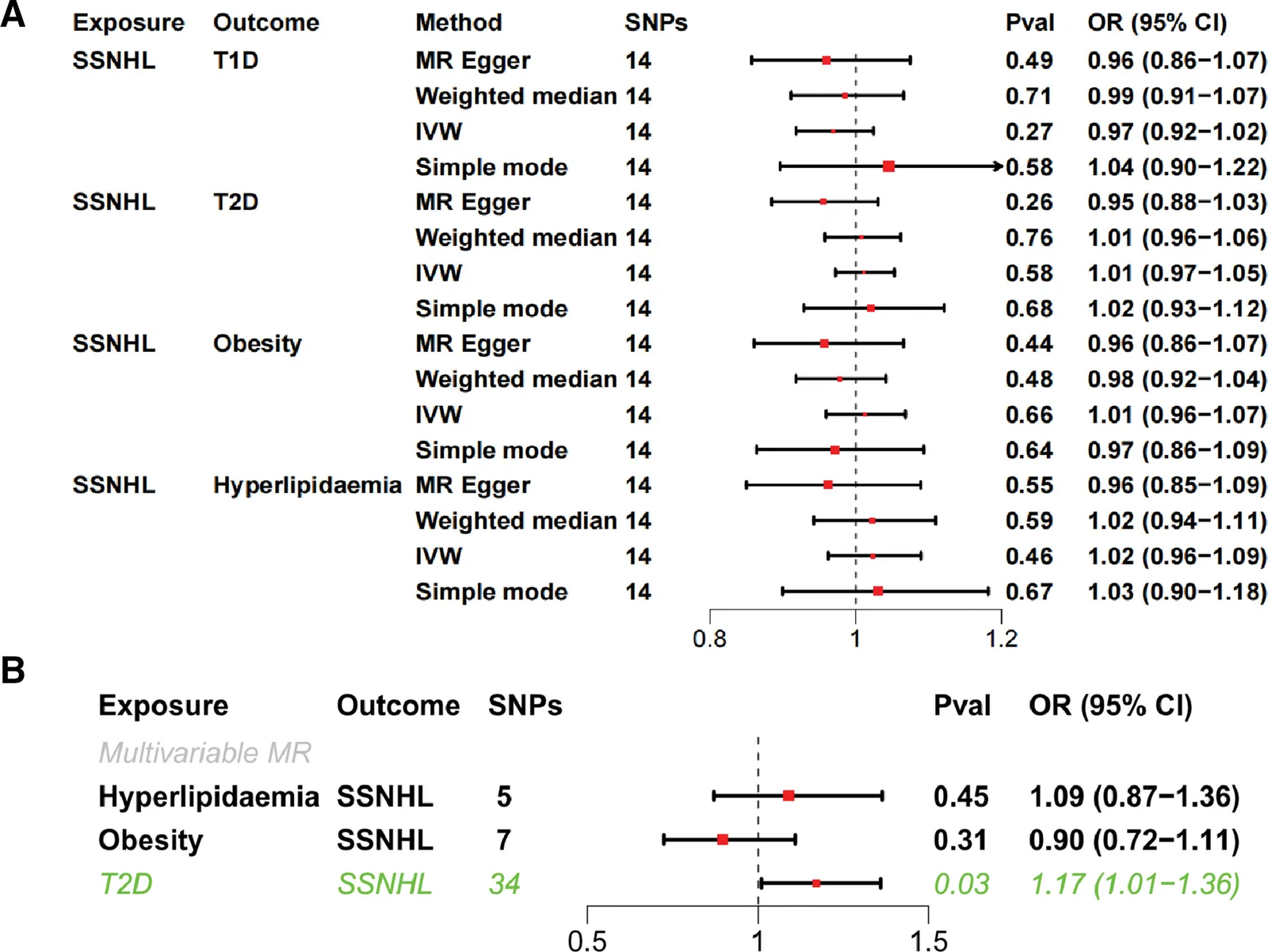
Reverse MR and multivariate MR. (A) Reverse univariate MR analysis of the causal relationship between diabetes, obesity, hyperlipidemia, and SSNHL. (B) Multivariate MR analysis of the causal relationship between diabetes, obesity, hyperlipidemia, and SSNHL. CI = confidence interval, IVW = inverse variance weighted, MR = Mendelian randomization, OR = odds ratio, SNP = single-nucleotide polymorphism, SSNHL = sudden sensorineural hearing loss, T1D = type 1 diabetes, T2D = type 2 diabetes.

All IVs demonstrated strong instrument validity (mean *F*-statistic = 27.72, range = 22.22–36.53), effectively mitigating weak instrument bias. After assessing and removing SNPs associated with confounding, the results of univariate MR analysis are shown in Figure 2. The MR analysis conducted using the IVW method reveals a genetic causal relationship between T2D and the risk of SSNHL (odds ratio [OR] = 1.18, 95% confidence interval [CI] = 1.02–1.36, *P* = .03). The forest plot illustrates that genetically predicted T2D is significantly associated with SSNHL (Fig. 3A). The MR analysis conducted using the weighted median method also yielded similar results (OR = 1.22, 95% CI = 1.00–1.48, *P* = .05). Similarly, risk estimates from MR-Egger regression and simple mode methods exhibited similar trends, although these associations did not reach statistical significance (Fig. 3B). The results of heterogeneity and horizontal pleiotropy analysis are shown in Table 1. *P* values obtained from the Cochran *Q* tests for MR-Egger (Cochran *Q* = 40.44, *P* = .14) and IVW (Cochran *Q* = 41.75, *P* = .14) were >.05, indicating no heterogeneity in the results (Fig. 3C). The global test for MR-PRESSO (*P* global test = .13) indicated that no anomalous IVs contributed to the effect of multiplicity in the overall MR estimates. Leave-one-out sensitivity analyses affirmed the robustness of the conclusion (Fig. 3D). However, we found no evidence supporting a causal relationship between T1D (IVW, OR = 1.05, 95% CI = 0.99–1.10, *P* = .06), obesity (IVW, OR = 0.95, 95% CI = 0.77–1.17, *P* = .61), hyperlipidemia (IVW, OR = 1.14, 95% CI = 0.86–1.52, *P* = .36), and SSNHL. After applying the aforementioned criteria, we identified 14 SNPs significantly associated with SSNHL (Table S1, Supplemental Digital Content, https://links.lww.com/MD/R616). In our reverse MR analysis using the IVW method, no significant evidence supporting a causal relationship between SSNHL and the risk of T2D, T1D, obesity, and hyperlipidemia was found (Fig. 4A).

**Table 1:** Pleiotropy and heterogeneity test of T1D, T2D, obesity, hyperlipidemia in GWAS for SSNHL.

Expose	MR-Egger intercept test		Cochran *Q* test				MR-PRESSO	
	Egger_intercept	Pval	*Q* (IVW)	*Q*_Pval	*Q* (MR-Egger)	*Q*_Pval	Outlier	Pval
T1D	−0.0469	.06	11.5	.72	7.13	.95	14.16	.72
T2D	0.0215	.27	41.75	.14	40.44	.14	39.08	.13
Obesity	−0.0607	.26	3.19	.86	1.66	.94	5.61	.78
Hyperlipidemia	−0.1218	.10	6.8	.14	1.61	.64	10.57	.21

GWAS = genome-wide association studies, MR = Mendelian randomization, SSNHL = sudden sensorineural hearing loss, T1D = type 1 diabetes, T2D = type 2 diabetes.

### 3.2. Multivariate MR analysis of the causal relationship between T2D, obesity, hyperlipidemia, and SSNHL

Given the substantial body of prior research indicating that obesity and hyperlipidemia are common risk factors for both T2D and SSNHL, we conducted an MVMR analysis including T2D, obesity, and hyperlipidemia as exposures to investigate the direct impact of T2D on SSNHL. The results of the MVMR analysis indicate that even after adjusting for obesity and hyperlipidemia, results consistent with the univariate MR analysis were attained (T2D, OR = 1.17, 95% CI = 1.01–1.36, *P* = .03; Fig. 4B). These results once again confirm that T2D is a potential risk factor for SSNHL. This finding further underscores that the genetic evidence is consistent with a modest causal effect of T2D on SSNHL risk. We assessed instrument strength and multicollinearity in our MVMR analysis. The conditional *F*-statistics were 4.43 for hyperlipidemia, 10.12 for obesity, 33.43 for T1D, and 15.35 for T2D, indicating adequate to strong instruments for all exposures except hyperlipidemia, which showed weak instrument strength. Concurrently, all variance inflation factors were below 1.7 (T1D = 1.51, hyperlipidemia = 1.13, obesity = 1.05, T2D = 1.67), demonstrating minimal multicollinearity. These results support the validity of our MVMR estimates while highlighting the need for cautious interpretation of the hyperlipidemia findings due to potential weak instrument bias.

## 4. Discussion

Based on comprehensive large-scale GWAS meta-analyses, we utilized bidirectional MR and MVMR analyses to investigate the causal relationships between diabetes, obesity, hyperlipidemia, and SSNHL. Our results indicate a positive correlation between T2D and the risk of SSNHL. However, there is insufficient evidence to support significant associations between T1D, obesity, hyperlipidemia, and SSNHL risk. Further multivariate MR analysis suggests that the impact of T2D on SSNHL persists even after considering risk factors such as obesity and hyperlipidemia.

Current epidemiological evidence regarding the association between diabetes and SSNHL remains inconsistent. One retrospective cohort study reported an SSNHL incidence of 1.29 per 1000 among diabetic patients, representing a 1.54-fold increase compared with nondiabetic individuals.^[10]^ Aimoni et al further demonstrated a positive correlation between diabetes and SSNHL risk.^[20]^ However, studies by Jalali et al^[21]^ and Rajati et al^[35]^ found no statistically significant difference in SSNHL incidence between diabetic and nondiabetic populations. These discrepancies may stem from methodological variations across studies, particularly the failure to distinguish between diabetes types. Notably, a systematic review and meta-analysisconducted by Akinpelu et al,^[36]^ stratified by diabetes type, revealed that only T2D constitutes an independent risk factor for SSNHL, a conclusion consistent with our findings. Our findings suggest that T2D may be a potential risk factor for SSNHL, possibly mediated by several interconnected pathophysiological pathways. First, T2D is known to induce systemic microvascular dysfunction, characterized by capillary basement membrane thickening and impaired endothelial function.^[37]^ Within the inner ear, the highly vascularized stria vascularis forms the blood-labyrinth barrier, playing a crucial role in maintaining the unique electrochemical environment of the organ of Corti.^[38,39]^ This structure also generates the endolymphatic potential and cochlear potential.^[40]^ The transient hypoxia-induced reduction in cochlear endocochlear potential and accompanying hearing loss underscore the energy demands required to sustain the cochlear microenvironment.^[41]^ Diabetic microvascular impairment can compromise cochlear blood flow, oxygen exchange, and ion transport efficiency.^[42]^ Animal models of diabetes associated with cochlear microvascular pathology demonstrate structural alterations, including strial degeneration, loss of cochlear sensory receptors, and changes in neural fiber architecture.^[43]^ Furthermore, temporal bone studies from diabetic patients reveal significant vascular dilation within the cochlear turns and the vascular walls of the basilar membrane. These patients also exhibit significantly greater outer hair cell loss in the cochlea, although spiral ganglion cell and inner hair cell counts remain unchanged.^[44]^ Collectively, this evidence suggests that diabetic microangiopathy may disrupt cochlear blood flow and blood-labyrinth barrier integrity, potentially leading to inner ear ischemia and ionic imbalance. Second, hyperglycemia-driven oxidative stress represents another hallmark of T2D.^[45]^ Excessive reactive oxygen species can directly damage cochlear hair cells and spiral ganglion neurons, which exhibit particular vulnerability to metabolic insult.^[46]^ Finally, T2D is characterized by a chronic inflammatory state, accompanied by elevated levels of inflammatory cytokines such as tumor necrosis factor-α and interleukin-6.^[13]^ The high expression of these inflammatory factors is considered one of the significant causes of the occurrence and progression of SSNHL.^[47]^ Critically, these pathways – microvascular dysfunction, oxidative stress, and inflammation – are not mutually exclusive but likely act synergistically to disrupt the delicate cochlear microenvironment, potentially precipitating SSNHL. Although our study provides genetic evidence supporting a causal relationship between T2D and SSNHL, the underlying molecular and structural mechanisms require further validation. Future experimental studies are necessary to directly observe and quantify these pathological changes in the diabetic inner ear to verify the mechanistic framework proposed herein.

High cholesterol levels and/or elevated triglycerides, characteristic of hyperlipidemia, represent another risk factor for SSNHL.^[47]^ Hyperlipidemia disrupts blood flow through processes such as plaque formation, vascular remodeling, and vessel lumen obstruction. This can lead to endothelial dysfunction and vascular inflammation while also increasing the accumulation of lipids and cholesterol on the vascular wall endothelium. This disruption ultimately compromises the oxygen supply to target organs such as the cochlea.^[48,49]^ Previous extensive studies have shown that compared with the control group, patients with SSNHL exhibit significantly elevated levels of plasma cholesterol, triglycerides, lipoprotein A, and low-density lipoprotein cholesterol concentrations.^[11,50]^ Therefore, hyperlipidemia is considered a risk factor for SSNHL, although conflicting evidence exists, rendering the association between hyperlipidemia and SSNHL uncertain.^[22]^ To date, there has been limited research analyzing the association between obesity and SSNHL. Atherosclerosis related to obesity may lead to arteriosclerosis and constriction of the internal auditory artery, resulting in reduced cochlear blood flow. This process may ultimately lead to hearing loss through the induction of vascular stria and cell death.^[51]^ A study by Chien et al found that obesity is an independent risk factor for SSNHL.^[52]^ Researchers also observed significantly elevated auditory brainstem response thresholds in obese animal models.^[52]^ However, a study by Hwang provides retrospective cohort data on the effect of obesity on adult SSNHL recovery. The results indicate no correlation between obesity and SSNHL recovery.^[23]^ As metabolic disorders are complex conditions encompassing known factors that may affect the onset and prognosis of SSNHL, such as diabetes, hyperlipidemia, and obesity, the association between metabolic disorders and SSNHL may result from a single factor or the combined effects of these factors. Utilizing combined bidirectional MR and multifactorial MR can minimize confounding effects to the greatest extent, thereby yielding reliable and robust analytical outcomes.

This study design offers notable advantages. First, it leverages freely accessible GWAS data, significantly reducing research costs. Second, employing genetic variants as IVs in MR analysis effectively mitigates confounding biases and reverse causation effects. Last, our findings demonstrate a potential association between T2D and increased risk of SSNHL, thereby providing potential clinical preventive strategies for otologists. However, acknowledging certain potential limitations in our study is necessary. First, the use of GWAS data primarily from individuals of European ancestry precludes generalizability of our findings to other populations. Second, the statistical power of our analysis was limited by the sample size of the SSNHL GWAS. This, in conjunction with the established clinical heterogeneity of SSNHL, may lead to an underestimation of causal effects and could bias our estimates, which may explain some of our null findings. Third, although SSNHL comprises distinct subtypes, subgroup-specific MR analyses were precluded by the lack of sufficiently detailed GWAS data. Consequently, our results represent an aggregate effect across a heterogeneous phenotype, and future investigations with larger, well-phenotyped cohorts are needed to dissect etiology-specific mechanisms.

In summary, our bidirectional MR and MVMR analyses demonstrate that T2D may be a potential risk factor for the onset of SSNHL. However, we found no genetic evidence of independent causal relationships between T1D, obesity, hyperlipidemia, and SSNHL risk. These findings contribute to our understanding of the relationship between diabetes and SSNHL, emphasizing the potential associations between different types of diabetes and SSNHL. We anticipate that our research results will inform otologists about clinical prevention and treatment strategies for SSNHL.

## Acknowledgments

The authors thank the FinnGen study in our analysis for providing a publicly available GWAS dataset.

## Author contributions

**Conceptualization:** Sufen Peng, Chunmei Lu, Li Lin, Chenghong Guo.

**Data curation:** Sufen Peng, Chunmei Lu, Jialei Chen.

**Investigation:** Sufen Peng, Chunmei Lu, Jialei Chen.

**Methodology:** Sufen Peng, Chunmei Lu, Li Lin, Chenghong Guo.

**Resources:** Sufen Peng, Chunmei Lu, Chenghong Guo.

**Visualization:** Sufen Peng, Chunmei Lu.

**Funding acquisition:** Li Lin.

**Project administration:** Li Lin, Chenghong Guo.

**Writing – original draft:** Sufen Peng, Chunmei Lu, Li Lin, Jialei Chen, Chenghong Guo.

**Writing – review & editing:** Sufen Peng, Chunmei Lu, Li Lin, Jialei Chen, Chenghong Guo.

## Supplementary Material


